# B-Cell Chronic Lymphocytic Leukemia with 11q22.3 Rearrangement in Patient with Chronic Myeloid Leukemia Treated with Imatinib

**DOI:** 10.1155/2016/9806515

**Published:** 2016-03-03

**Authors:** Krzysztof Lewandowski, Michał Gniot, Maria Lewandowska, Anna Wache, Błażej Ratajczak, Anna Czyż, Małgorzata Jarmuż-Szymczak, Mieczysław Komarnicki

**Affiliations:** ^1^Department of Hematology and Stem Cell Transplantation, Poznan University of Medical Sciences, Szamarzewskiego 82/84, 60-569 Poznan, Poland; ^2^Institute of Human Genetics, Polish Academy of Sciences, Strzeszyńska 32, 60-479 Poznan, Poland

## Abstract

The coexistence of two diseases chronic myeloid leukemia (CML) and B-cell chronic lymphocytic leukemia (B-CLL) is a rare phenomenon. Both neoplastic disorders have several common epidemiological denominators (they occur more often in men over 50 years of age) but different origin and long term prognosis. In this paper we described the clinical and pathological findings in patient with CML in major molecular response who developed B-CLL with 11q22.3 rearrangement and Coombs positive hemolytic anemia during the imatinib treatment. Due to the presence of the symptoms of autoimmune hemolytic anemia and optimal CML response to the imatinib treatment, the decision about combined therapy with prednisone and imatinib was made. During the follow-up, the normalization of complete blood count and resolution of peripheral lymphadenopathy were noted. The hematologic response of B-CLL was diagnosed. The repeated FISH analysis of cultured peripheral blood lymphocytes showed 2% of cells carrying 11q22.3 rearrangement. At the same time, molecular monitoring confirmed the deep molecular response of CML. The effectiveness of such combination in the described case raises the question about the best therapeutic option in such situation, especially in patients with good imatinib tolerance and optimal response.

## 1. Introduction

Chronic myeloid leukemia (CML) and chronic B-cell lymphocytic leukemia (B-CLL) are the most common hematological diseases in adult people. They occur with the frequency of about 1 per 100,000 for CML and 4 per 100,000 for B-CLL. The coexistence of CML and B-CLL is a rare phenomenon. Both diseases have several common epidemiological denominators. Firstly, they occur more often in men. Secondly, both disorders occur more frequently in people over 50 years of age. In CML, the acquisition of genetic defect translates to the modification of the life cycle of the stem cell resulting in abnormal proliferation and differentiation, genomic instability, and abnormal apoptosis [1]. The pathogenesis of B-CLL is definitely less understood. Malignancy originated from mature B lymphocytes which are highly dependent on interactions with the tissue microenvironment in terms of their survival and proliferation. Also, the acquisition of cytogenetic and molecular aberrations by malignant cells plays an important role [2, 3]. Despite different clinical and pathological characteristics, the medical databases describe only over 20 cases of the coexistence of the CML and B-CLL [4]. Most reported cases refer to the situation when the diagnosis of CML was preceded by the diagnosis of CLL. Relatively fewer cases documented the coexistence of CML and B-CLL simultaneously. Definitely the rarest situation refers to the occurrence of CLL during the CML treatment. The present clinical case is based on a systematic review of the literature available in the PubMed database (June 2015).

## 2. Case Report

A 68 year-old man was admitted to hospital in March 2014 with the suspicion of blast crisis of chronic myeloid leukemia. The diagnosis of CML was established in 2001. Initially, to reduce tumor mass, he was treated with hydroxyurea in a dose of 50 mg/kg/day orally. Thereafter, from June 2001 to June 2002, interferon-*α* in a dose of 5 mln U/m^2^ daily subcutaneously in combination with cytosine arabinoside (20 mg/m^2^/subcutaneously daily) was given. Routine treatment efficacy evaluation performed in October 2003 confirmed complete hematologic (CHR) and complete cytogenetic remission (CCyR). In November 2004, the loss of CHR was diagnosed. Leukocytes count in the peripheral blood increased up to 15,0 G/L. Peripheral blood white blood cell differential revealed granulocytes at all stages of maturation with 6% of myeloblasts. FISH analysis confirmed the presence of the Ph chromosome in 35% interphase nuclei in the peripheral blood. Due to the loss of CHR, therapy with imatinib (IM) in a dose of 400 mg/day was initiated in February 2005.

**Figure 1:**
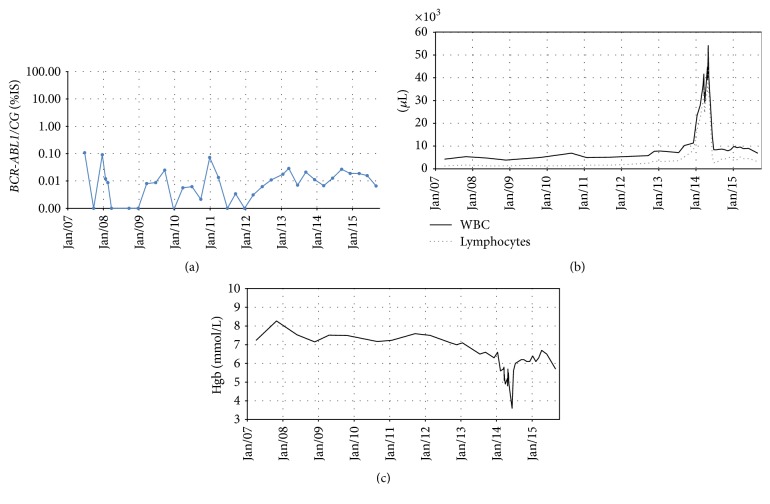
The results of the laboratory tests performed during the patient's follow-up. (a) Molecular response analysis based on the* BCR-ABL* transcript level evaluation on International Scale. (b) Peripheral blood leukocyte (black line) and absolute lymphocytes (gray dots) count during the observation time when the patient remained in CCyR and MMR. (c) Blood hemoglobin (Hgb) concentration during IM treatment. A significant drop in Hgb level was noted simultaneously with the rise of absolute lymphocyte blood count.

After 3 and 6 months of the treatment, the CHR and CCyR were reobtained, respectively. The major molecular response (MMR) was documented in January 2008. The kinetics of molecular response and peripheral blood hemoglobin level and leukocyte/lymphocyte count are shown in Figure 1. During 9 years of follow-up, the IM treatment was well tolerated, without any significant hematological and nonhematological toxicity. The monthly clinical and laboratory evaluation, as well as the IM uptake on the basis of clinical interviews, self-reporting, and prescribed pill counts over a 30-day period, did not show any abnormalities.

**Figure 2:**
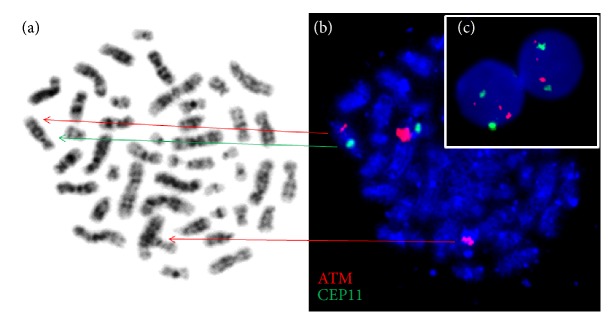
The patient's bone marrow cell culture. (a) Metaphase staining by Giemsa contains alteration chromosomes 8 and 11 from translocation t(?8;11)(q?13;q22) (indicated by arrows). (b) FISH results with ATM/CEP11 probe on the same metaphase showed rearrangement in 11q22.3 band (two smaller red signals and one normal signal). (c) Interphase FISH results with specific probe ATM/CEP11 reveal three signals (one normal and two smaller signals) for ATM and two typical signals for CEP 11.

In January 2014, routine complete blood count analysis revealed that leukocytosis with an increase of absolute lymphocytes count (ALC) up to 26,3 G/L slightly decreased hemoglobin level and normal platelets count. At this time, the patients remained in MMR and did not complain about fever, weight loss, night sweats, and bone pains. Detailed clinical evaluation performed in April 2014 documented the presence of peripheral, mediastinal, and abdominal lymphadenopathy. Subsequent peripheral blood analysis showed anemia (Hb 5,8 mmol/L) and leukocytosis (WBC 41,65 G/L) with ALC 35,3 G/L and normal platelets count 343,0 G/L. Direct antiglobulin test was positive. However, absolute reticulocyte count 61,3 G/L and bilirubin concentration in the blood was normal. Lactic dehydrogenase activity (294 U/mL) was slightly elevated. The blood smear had an increase in small, normally appearing lymphocytes with the presence of smudge cells. The bone marrow (BM) aspiration biopsy confirmed massive infiltration of bone marrow by lymphocytic cells resembling small lymphocytes (accounted for 90% of all nucleated bone marrow cells). The immunophenotypic evaluation of the CD45+ BM mononuclear cells revealed the presence of a monoclonal population of B lymphocytes expressing CD5/CD19 and CD23. Trephine biopsy showed hypercellular bone marrow with massive infiltration by small lymphocytic cells (about 80%). Erythroid, granulocytic, and megakaryocytic cells BM content was lower than normally, but their maturation was normal. Fibrotic changes were visible in 90% of the microscopic viewing area (MF1/2). On the basis of clinical and laboratory data, B-cell chronic lymphocytic leukemia was diagnosed. The routine karyotype cytogenetic analysis of BM cells performed in March 2014 showed 46,XY,t(?8;11)(q?13;q22)[1]/46,XY[28]. Interphase FISH study of the BM cells with the use of a locus specific probe for* ATM* [del(11)(q22.3)] and CEP11 (Vysis, Abbott Molecular Inc., Des Plaines, IL, USA) documented the abnormal signal for ATM locus (two smaller signals and one normal signal) in the malignant cells in 72% interphase nuclei. The results of FISH study with the use of molecular probes for 13q14 (D13S19)/13q34 (LAMP1)/CEP12 (Vysis, Abbott Molecular Inc., Des Plaines, IL, USA) and [del(17)(p13.1)] probe for* TP53* did not reveal any additional aberrations. Retrospective analysis of patient samples collected in April 2009 and April 2012, before the occurrence of B-CLL symptoms, confirmed the absence of the rearrangement of 11q22.3. However, the same anomaly of chromosome for 11q22.3 band was found in 46% of BM cells in the sample collected in April 2013 (Figure 2). Repeated FISH analysis performed in the March 2015 showed rearrangement of 11q22.3 in 53% of bone marrow interphase nuclei. The classical cytogenetic study of BM cells confirmed the absence of translocation t(9;22).

Due to the presence of the symptoms of autoimmune hemolytic anemia, the decision about combined treatment with IM and steroids (prednisone 1 mg/kg b.w. orally daily) was made. During the follow-up, the resolution of peripheral lymphadenopathy, reduction of ALC, and significant improvement in the hemoglobin level were noted (Figure 1). Therefore, prednisone daily dose was reduced to 15 mg/day. The hematologic response of B-CLL was diagnosed in January 2015. The repeated FISH analysis of cultured peripheral blood lymphocytes performed 8 months later showed 2% of cells carrying with 11q22.3 rearrangement. At the same time, molecular monitoring confirmed the deep molecular response of CML.

## 3. Discussion

The coexistence of two or more neoplasms in patients with hematologic malignancy is a relatively rare situation. In the available literature, the risk of secondary tumor in CML is increased by 50%. Similar standardized incidence ratios before and after the second year following the diagnosis may indicate that this phenomenon is linked to the CML disease itself, rather than the tyrosine kinase inhibitor treatment [5]. It has been also proven that, among patients diagnosed with CLL, the risk of secondary cancers increases [6]. This phenomenon is being explained by treatment with alkylating agents and/or by the impaired immune function. But the rarest scenario is applied to situations when the CLL occurs in patients with previously diagnosed CML. One of the proposed explanations assumes that in this case the* BCR-ABL*-positive cells produce a number of cytokines, including IL-3, which stimulates the production of progenitor B lymphocytes [7].

In a recently published report, Olcaydu et al. estimated that the frequency of CLL in patients with familial and sporadic myeloproliferative neoplasms (MPN) was relatively high (10 and 2%, resp.). The authors also noticed that patients with familial clustering of MPN might harbor a yet unidentified predisposition to develop malignant disorders in general, independently of the* JAK2* haplotype [8]. In case of CML, there is contradictory evidence regarding hereditary risk of the disease development. Björkholm et al. did not reveal any significant familial aggregation in an analysis based on the Swedish Cancer Registry [9], but, on the other hand, Li et al. suggest that some gene polymorphisms may increase the risk of CML [10].

Genetic susceptibility to chronic lymphocytic leukemia was suggested by Slager et al. [11]. Despite the genetic predispositions to CLL and CML [12], the diseases also do not share a common pattern of cytogenetic and molecular abnormalities. In a sporadic form of CLL, the deletions of 11q, 13q, 17p, and chromosome 12 trisomy have a documented prognostic value and play an important role in the CLL pathogenesis and disease evolution [13]. Moreover, genomic arrays revealed the presence of other small recurrent submicroscopic abnormalities—deletions in 22q11 and gains of 20q13.12 which were detected in 15% and 19% of the CLL patients, respectively. Both abnormalities showed related gene expression changes, revealing the high diversity of genomic aberrations in CLL [14, 15]. In CML, subtle genetic changes are relatively common, including somatic segmental copy number changes (i.e., the loss of 9q34, 15q25.3, and 15q13 and a gain of 7p21.1-p15.3) [16]. As far as the diagnosis of CML is concerned, the presence of additional clonal cytogenetic aberrations may be observed in 5% of the CML patients in the chronic phase. Their appearance is mostly associated with the disease evolution and their frequency is higher in the late chronic phase, acceleration phase (30%), and blast crisis (80%) [17].

In the CLL patients, chromosome 12 trisomy is considered a clonal driver alteration that occurs early in the disease evolution and facilitates the appearance of secondary chromosomal aberrations or mutations in genes as* NOTCH1*,* TP53*, and* FBXW7* [18, 19]. Similarly, in the myeloid blast crisis of CML, the most common mutations occur at the loci of the* TP53* tumor suppressor gene (20%–30% cases) and the runt-related transcription factor gene (*RUNX1*, 38% of cases). In the lymphoid blast crisis, the most frequent mutations can be found in the cyclin-dependent kinase inhibitor 2A/2B gene (*CDKN2A/B*, 50%) and Ikaros transcription factor (*IKZF1*, 55%) [20]. Lastly, antagonistic relationship and cross-talk between BCR-ABL and NOTCH1 activity (which are critical for hematopoietic stem cell self-renewal and survival) in CML progenitor cells were noticed, however, their significance in coexisting CML and CLL remains unclear [21].

A very interesting report was published by Fattizzo et al. [22]. Authors reported an unusual case of three hematological malignancies in the same patient: CLL, CML, and acute myeloid leukemia (AML). According to their opinion, none of the three malignancies shared the same origin, since the marrow sample was negative for the* BCR-ABL1* transcript at the time of the CLL diagnosis, CLL was in remission at the CML diagnosis, and CML was in complete cytogenetic response at the AML symptoms onset, indicating that this was not a blast crisis. However, it should be kept in mind that in our and other cases there is a possibility of a therapy-induced secondary malignancy [23].

Another question is the relation between acquisition of specific genetic defect(s) and the appearance of symptoms of B-CLL or CML in patients with CML or B-CLL, respectively. In 2009, Tecchio et al. [24] documented successful clinical outcome of a previously untreated B-CLL patient (trisomy 12 positive, IgVH mutated, and ZAP-70 negative) who experienced CML nine years after. Dasatinib (100 mg/day) was introduced after 2 months of initial treatment with IM due to its unacceptable toxicity. Therapy resulted in CHR and deep molecular response. As far as B-CLL is concerned, the improvement was manifested by the disappearance of the lymphadenopathy, reduction of spleen size, drop in ALC up to 8%, and reduction of trisomy 12 positive cells content in peripheral blood up to 7%. Similar data concerning a CML patient who developed B-CLL with 11q22 deletion at* ATM* locus in 4th month of successful IM treatment was published by Serpa et al. in 2010. According to the literature data suggesting the effectiveness of dasatinib on CLL, the authors replaced IM with dasatinib. The switch to dasatinib allowed obtaining partial response, characterized by the regression of lymph node enlargement and significant reduction of lymphocytosis [25]. Similar favorable outcome of B-CLL was observed in our patient with CML and MMR on IM treatment when prednisone treatment was given. Detailed mechanisms of proapoptotic action of prednisone in this clinical situation are not well understood. Glucocorticosteroids impose metabolic stress on B-CLL cells by altering metabolic gene expression and activity in CLL cells, prevention of tumor cells from accessing bioenergetic programs required to respond to membrane damage, and increasing the dependence of CLL cells on fatty acid oxidation by altering the expression of* PPARa* and* PDK4* [26]. The use of IM raises another question in our case. Since 2004, the patient was treated with IM in a dose 400 mg orally daily. Retrospective cytogenetic analysis showed that the* ATM* rearrangement was already present in 2013 (in BM), when the patient was in deep molecular response (Figure 1). According to the available data, dasatinib induces apoptosis of CLL cells* in vitro* [27]. Veldurthy et al. suggest that this effect is associated with dasatinib's inhibitory activity not only against BCR-ABL but also against SFK (*Src Family Kinases*) which may imply that this TKI should be considered in the situation of CML and B-CLL coexistence.

In our opinion, the key to understanding the striking response observed in our patient after addition of prednisolone to IM is probably due to the inhibition by such combination of Lck kinase. According to both* in vitro* and* in silico* data, LCK kinase can be inhibited not only by dasatinib but also by imatinib [28, 29]. Despite the fact that IC50 of IM towards Lck is approximately 40 times higher than Abl, it still can be considered to be a moderately effective Lck inhibitor. It was confirmed that the activity of Lck kinase protects CLL cells from glucocorticoid-induced apoptosis [30]. This could imply that Lck inhibition may resulted in an increased apoptosis of leukemic cells [29, 30]. The latter phenomenon was documented in case of T-CLL [30, 31]. Our clinical observations could mean that similar effect is also possible in case of B-CLL. Therefore, in our opinion the combination of IM with prednisolone might be a good therapeutic option in CML patients with coexisting B-CLL, especially when IM treatment tolerance is good and response is optimal.

## 4. Conclusion

Patients with CML should be carefully monitored for the presence of secondary neoplasms, even when they respond optimally to the tyrosine kinase treatment. Decision about antineoplastic treatment should be based on the type of the secondary tumor and possible beneficial drugs interactions.
